# *FON2 SPARE1* Redundantly Regulates Floral Meristem Maintenance with *FLORAL ORGAN NUMBER2* in Rice

**DOI:** 10.1371/journal.pgen.1000693

**Published:** 2009-10-16

**Authors:** Takuya Suzaki, Masako Ohneda, Taiyo Toriba, Akiko Yoshida, Hiro-Yuki Hirano

**Affiliations:** Department of Biological Sciences, Graduate School of Science, University of Tokyo, Bunkyo-ku, Tokyo, Japan; The University of North Carolina at Chapel Hill, United States of America

## Abstract

CLAVATA signaling restricts stem cell identity in the shoot apical meristem (SAM) in *Arabidopsis thaliana*. In rice (*Oryza sativa*), FLORAL ORGAN NUMBER2 (FON2), closely related to CLV3, is involved as a signaling molecule in a similar pathway to negatively regulate stem cell proliferation in the floral meristem (FM). Here we show that the *FON2 SPARE1* (*FOS1*) gene encoding a CLE protein functions along with *FON2* in maintenance of the FM. In addition, *FOS1* appears to be involved in maintenance of the SAM in the vegetative phase, because constitutive expression of *FOS1* caused termination of the vegetative SAM. Genetic analysis revealed that FOS1 does not need FON1, the putative receptor of FON2, for its action, suggesting that FOS1 and FON2 may function in meristem maintenance as signaling molecules in independent pathways. Initially, we identified FOS1 as a suppressor that originates from *O. sativa indica* and suppresses the *fon2* mutation in *O. sativa japonica*. FOS1 function in *japonica* appears to be compromised by a functional nucleotide polymorphism (FNP) at the putative processing site of the signal peptide. Sequence comparison of *FOS1* in about 150 domesticated rice and wild rice species indicates that this FNP is present only in *japonica*, suggesting that redundant regulation by FOS1 and FON2 is commonplace in species in the *Oryza* genus. Distribution of the FNP also suggests that this mutation may have occurred during the divergence of *japonica* from its wild ancestor. Stem cell maintenance may be regulated by at least three negative pathways in rice, and each pathway may contribute differently to this regulation depending on the type of the meristem. This situation contrasts with that in *Arabidopsis*, where CLV signaling is the major single pathway in all meristems.

## Author Summary

The body plan of plants is regulated by the function of apical meristems that are generated in the embryo. Leaves and floral organs are derived from cells supplied by stem cells in the vegetative shoot apical meristem (SAM) and the floral meristem (FM), respectively. Thus, genetic regulation of stem cell maintenance is a central issue in plant development. In the model plant *Arabidopsis thaliana*, CLAVATA3 (CLV3) functions as a key signaling molecule to restrict the size of the stem cell population in both the SAM and the FM. In rice, however, we show here that two *CLV3*-like genes, *FLORAL ORGAN NUMBER2* (*FON2*) and *FON2 SPARE1* (*FOS1*), redundantly regulate maintenance of the FM. We also show that *FOS1* is likely to be involved in maintenance of the vegetative SAM, whereas *FON2* plays no role in regulation in this meristem. FOS1 appears to act via a putative receptor that differs from the FON2 receptor, suggesting that these two signaling molecules function in independent pathways to restrict stem cells in different ways depending on the type of meristem. In addition, we show that the *FOS1* gene was compromised in the standard rice, *Oryza sativa spp. japonica*, during the evolution of rice.

## Introduction

Intercellular communication plays a crucial role in the determination of cell fate in plant development. Cell fate is determined by positional information emanating from neighboring or distant cells. Recent molecular genetic studies have revealed that peptide signaling molecules are involved in intercellular communication to regulate various aspects of plant development, such as stem cell maintenance, vascular differentiation, stomata patterning, and leaf size control [1]–[5].

The *CLE* genes encode small secreted proteins with a plant-specific domain called the CLE domain [6]. The *CLAVATA3* (*CLV3*) gene of *Arabidopsis thaliana*, and the *FLORAL ORGAN NUMBER2* (*FON2*) and *FON2-LIKE CLE PROTEIN1* (*FCP1*) genes of rice (*Oryza sativa*) are involved in stem cell maintenance in the shoot apical meristem (SAM) and the floral meristem (FM) [1],[7],[8]. Tracheary element differentiation inhibitory factor (TDIF) has a role in suppressing the differentiation of tracheary elements in *Zinnia elegans* [3]. Recent biochemical studies have revealed that functional peptides of CLV3 and TDIF in vivo are dodeca peptides derived from the conserved CLE domains [3],[4].

In *Arabidopsis*, stem cell identity in the SAM is maintained by a regulatory feedback loop comprising the *CLV* and *WUSCHEL* (*WUS*) genes [9],[10]. CLV3 acts as a negative regulator of stem cell maintenance by repressing *WUS*, which encodes a novel homeodomain transcription factor that is expressed in the organizing center and promotes the identity of the stem cells overlying its expression domain [9]–[11]. Conversely, *WUS* positively regulates *CLV3* expression in the stem cell region. CLV3 peptide secreted from the stem cell appears to act through putative receptor complexes, consisting of CLV1, CLV2 or CORYNE/SOL2 [1], [12]–[15]. A recent biochemical study has revealed that CLV3 peptide binds directly to the extracellular domain of CLV1 [16]. When negative regulation of CLV signaling is removed by severe mutations of the *CLV1* and *CLV3* genes, enlargement of the SAM and the FM occurs, resulting in a fasciated stem and an increase in the number of flowers and floral organs [17],[18].

A similar genetic mechanism to regulate stem cell maintenance seems to be conserved in monocots. Mutations in the *FON1* and *FON2* genes in rice cause enlargement of the FM, resulting in an increase in the number of floral organs such as stamens and carpels [7],[8],[19],[20]. A double mutant of *fon1* and *fon2* shows no additive phenotype, suggesting that the two genes act in the same genetic pathway [8]. *FON1* encodes a receptor-like kinase with a leucine-rich repeat (LRR) structure in the extracellular domain that is closely related to *Arabidopsis* CLV1 [7]. *FON2* is a member of the *CLE* gene family, and the CLE domain of FON2 is similar to that of CLV3 [8],[21]. Likewise, in maize (*Zea mays*), the *thick tassel dwarf1* (*td1*) gene encodes a CLV1-like receptor kinase, and the *fasciated ear2* (*fea2*) gene, like *Arabidopsis CLV2*, encodes an LRR protein that lacks a cytoplasmic domain [22],[23]. Loss of function of these genes results in enlargement of the inflorescence meristem (IM) and the FM, causing fasciation of the inflorescences and an increase in floral organ number in maize. Constitutive expression of the *FON2* gene results in a severe decrease in the number of flowers and floral organs, probably because of a reduction in the size of the IM and the FM in rice, resulting in a phenotype similar to the *wus* flower [8],[11]. The effect of *FON2* overexpression is not observed in the *fon1* mutant, suggesting that FON1 is a putative receptor of FON2. Thus, *CLV*-related genes negatively regulate stem cell proliferation in the reproductive meristems in both rice and maize, as they do in *Arabidopsis*.

Despite this conservation, meristems in the vegetative phase are not affected by mutations in these *CLV*-related genes in the grasses, unlike in *Arabidopsis* [7],[8],[22],[23]. In rice, constitutive expression of *FON2* does not affect meristem maintenance in the vegetative phase [8],[24]. We previously showed that *FCP1* is probably involved in stem cell maintenance in the vegetative SAM because constitutive expression of *FCP1* causes consumption of the SAM, similar to overexpression of *CLV3* in *Arabidopsis* [24]. This action of FCP1 is also observed in *fon1* mutants, suggesting that FCP1 requires a receptor other than FON1. Thus, it is likely that, depending on the type of meristem, two independent pathways negatively regulate stem cell maintenance in rice. In maize, expression of *td1* is excluded from the vegetative SAM [23]. Thus, meristem maintenance in the vegetative phase is regulated differently from that in the reproductive phase in the grasses.

During the positional cloning of *FON2*, we found that expressivity of the *fon2* mutation is markedly reduced in F_2_ plants from a cross between the *fon2* mutant (*O. sativa japonica*) and Kasalath (*O. sativa indica*) [8]. To explain this difference, we hypothesized that the *indica* genome might contain genes that suppress the *fon2* mutation. In this paper, we describe the isolation and characterization of a gene, named *FON2 SPARE1* (*FOS1*), that suppresses the *fon2* mutation. *FOS1* encodes a secreted protein with a CLE domain, and is expressed in the SAM, IM and FM. Genetic and molecular analyses indicate that *FOS1*, together with *FON2*, is likely to be involved in stem cell maintenance in the FM, in rice species in the *Oryza* genus including *O. sativa indica*; by contrast, *FOS1* function seems to be severely compromised in *O. sativa japonica*. In addition, *FOS1* is likely to be involved in maintenance of the SAM in the vegetative phase, because overexpression of *FOS1* caused the formation of abnormal shoots with a terminated meristem. Analysis of the *FOS1* sequence from a large number of domesticated and wild rice species reveals that a nucleotide substitution related to the function of *FOS1* may have occurred during divergence of the domesticated rice *O. sativa japonica* from its wild ancestor.

## Results

### Expressivity of the *fon2* mutation is reduced in F_2_ plants of a cross between *fon2-1* (*japonica*) and Kasalath (*indica*)

**Figure 1:**
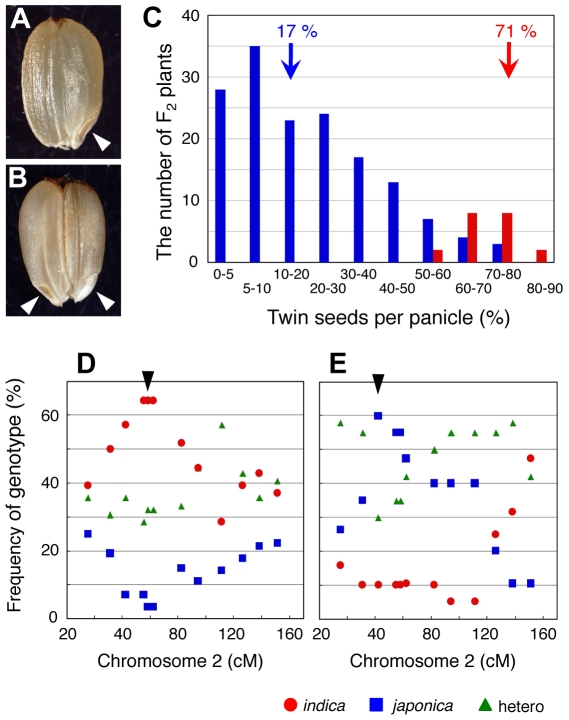
The *fon2* mutation is suppressed in F_2_ Progenies from a cross between *fon2* mutant (*japonica*) and Kasalath (*indica*). (A,B) Seed phenotype of wild type (A) and *fon2-1* mutant (B) of *japonica*. Arrowheads indicate embryos. (C) Reduction in expressivity in F_2_ plants from a cross between *fon2-1* and Kasalath. Blue bars indicate the distribution of F_2_ plants showing a *fon2* phenotype (n = 154). Red bars indicate the distribution of the *fon2-1* mutant (n = 20). Arrows indicate the median frequency in the appearance of the twin-seed phenotype per panicle. (D,E) Frequencies of genotypes of the molecular markers on chromosome 2 in F_2_ plants showing a *fon2* phenotype with high suppressing activity (twin seed frequency, <5%; n = 28) (D) or low suppressing activity (twin seed frequency, >45%; n = 20) (E). Arrowheads in (D,E) indicate the chromosomal position where high or low suppressing activity is closely associated with the genotypes homozygous for *indica* or *japonica*, respectively.

**Figure 2:**
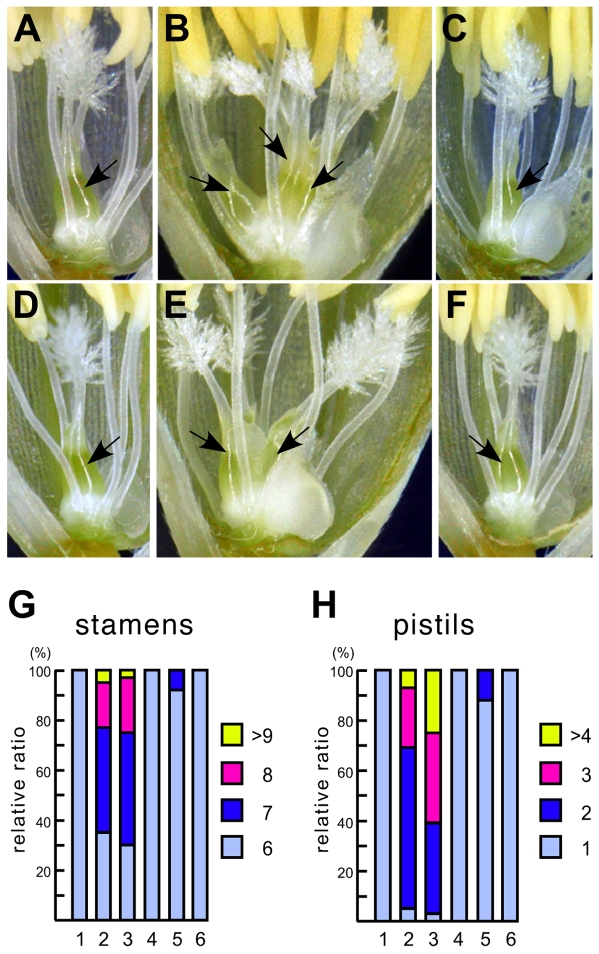
*Indica FOS1* suppresses the floral phenotypes caused by the *fon2* or *fon1* mutation. (A) A wild-type flower with one pistil in *japonica* (T65). (B) A *fon2-1* flower with three pistils. (C) A flower in *fon2-1* that is suppressed by *Agrobacterium*-mediated transformation of a genomic fragment containing the *FOS1* locus derived from *indica* (Kasalath). (D) A flower in a F_2_ plant from a cross between *fon2-1* and CSSL#9 that is homozygous for *fon2-1* and for the *FOS1* gene derived from Kasalath. The floral phenotype of *fon2-1* is suppressed by *indica FOS1*. (E) A *fon1-5* flower with two pistils. (F) A flower in *fon1-5* that is suppressed by *Agrobacterium*-mediated transformation of a genomic fragment containing the *FOS1* locus derived from *indica* (Kasalath). Arrows in (A–F) indicate pistils. (G) Number of stamens. (H) Number of pistils. 1, Wild type (T65); 2, *fon2-1*; 3, *fon1-5*; 4, *fon2-1* plant that is transformed with an *indica* (Kasalath) genomic fragment containing *FOS1*. 5, F_2_ plant from a cross between *fon2-1* and CSSL#9 that is homozygous for *fon2-1* and for *indica FOS1* derived from Kasalath. 6, *fon1-5* plant that is transformed with an *indica* (Kasalath) genomic fragment containing *FOS1*. For each strain, 100 flowers were examined.

In wild-type rice, a single pistil derived from congenitally fused carpels develops into a floret, and a single ovule is formed in the pistil. After fertilization, a single seed is formed within the husks, which are derived from the palea and lemma in a floret (Figure 1A). In *fon1* and *fon2* mutants, by contrast, the number of floral organs such as pistils increases due to an enlargement of the FM (Figure 2B) [8],[19]. Therefore, “twin seeds” are formed within the husks in these *fon* mutants (Figure 1B) when two or more pistils are produced in a floret (the third and fourth seeds cannot develop to maturity). Here we used the twin seed phenotype as an indication of *fon2* mutation.

In a previous screen of the *fon2* phenotype for positional cloning [8], we found that expressivity of the *fon2* mutation was reduced in F_2_ plants from a cross between the *fon2-1* mutant (*japonica*) and Kasalath (*indica*). To estimate quantitatively the frequency of the appearance of the *fon2* phenotype, we assessed the numbers of twin-seed phenotypes in this study. First, we counted the number of plants producing the twin-seed phenotype among F_2_ plants from the *fon2-1* and Kasalath cross. As a result, we found that the number of F_2_ plants showing a *fon2* phenotype was reduced markedly in the F_2_ plants: only 4.5% of F_2_ plants showed a *fon2* phenotype (Table 1). Second, we found that the number of the twin-seed phenotypes per panicle was also reduced markedly (Figure 1C). The median frequency of the appearance of the twin-seed phenotype per panicle was 71% in the *fon2-1* mutant (*japonica*); by contrast, it was reduced to 17% in the F_2_ plants showing a *fon2* phenotype. These results suggest that the *indica* (Kasalath) genome causes a reduction in the expressivity of the *fon2* mutation in the F_2_ progenies. In other words, it is likely that the *indica* genome has one or more genes that suppress the phenotype caused by the *fon2* mutation.

**Table 1:** Segregation ratio of the *fon2* phenotype in F_2_ plants from a *fon2-1* and Kasalath cross.

phenotype		ratio of mutants (%)
*fon2* phenotype *	wild type	
22	460	4.5

*If at least one twin seed per panicle was observed, the plant was considered as a mutant, and the genotype of the fon2 locus was confirmed by sequencing.

To address this possibility, we performed quantitative trait locus (QTL) analysis (see Materials and Methods). For QTL analysis, we checked the genotypes of the F_2_ plants showing the *fon2* phenotype to confirm that the *fon2* locus was homozygous for the mutation. As a result, a major QTL that suppressed the *fon2* mutation was detected in the region between 40 and 80 cM on chromosome 2. In a group of F_2_ plants showing high suppressor activity, the frequency of genotypes homozygous for the *indica* genome was very high, whereas that of genotypes homozygous for the *japonica* genome was very low (Figure 1D). In a group of F_2_ plants showing low suppressor activity, by contrast, the opposite result was obtained (Figure 1E). Thus, in this region of chromosome 2, high or low suppressor activity was closely associated with a genotype homozygous for *indica* or *japonica*, respectively.

### Identification of a gene that suppresses the *fon2* mutation in the *indica* genome

The above results indicated that a putative gene that suppresses the *fon2* mutation is located in the 40–80 cM region of chromosome 2 in the *indica* genome. We assumed that if *indica* has a gene that is functionally redundant to *FON2* in this region, this gene should behave as a suppressor-like gene function in genetic analyses. A strong candidate for such a gene would be a *CLE* gene, like FON2. A survey of rice genomic sequences identified a candidate *CLE* gene located at about 54 cM on chromosome 2.

Next, we examined whether the *CLE* gene on chromosome 2 in *indica* (tentatively named *CLE-C2*) could suppress the *fon2* mutation by conducting the following two experiments. First, we introduced a 3.3-kb genomic fragment from Kasalath containing the *CLE* gene into the *fon2-1* mutant by *Agrobacterium-*mediated transformation. We found that the defect in the *fon2* flowers was completely rescued in the transgenic plants, suggesting that the *CLE-C2* gene suppressed the *fon2* mutation (Figure 2C, 2G, and 2H). Second, we applied a genetic approach using a Nipponbare/Kasalath chromosomal segment substitution line (N/K CSSL#9), in which the chromosomal segment of Nipponbare (*japonica*) encompassing the *CLE-C2* gene was replaced by that of the Kasalath (*indica*) genome. We crossed *fon2-1* (*japonica*) with N/K CSSL#9, and then screened for F_2_ plants that were homozygous for both *fon2-1* and the *indica CLE-C2* allele by determining the genotype with molecular markers. The results indicated that the flower phenotype of the plants screened was identical to that of wild type, suggesting that the *fon2* mutation was also suppressed (Figure 2D, 2G, and 2H). Taken together, these results clearly indicate that the *CLE-C2* gene located at about 54 cM on chromosome 2 functions as a *fon2* suppressor. Thus, we designated this *CLE* gene as *FON2 SPARE1* (*FOS1*) because this gene can substitute for *FON2.*

### Molecular characterization of *FOS1*

**Figure 3:**
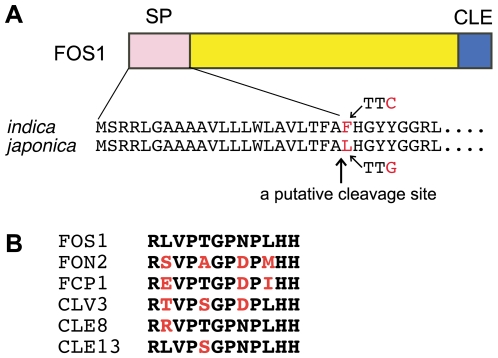
Characteristics of FOS1 protein. (A) Schematic representation of FOS1. The putative cleavage site of the signal peptide was predicted by signalP (http://www.cbs.dtu.dk/services/SignalP; Fletcher et al. 1999). Partial amino acid sequences of *japonica* and *indica* FOS1 are shown. (B) Alignment of amino acids from putative CLE peptides from related CLE proteins in rice (FOS1, FON2 and FCP1) and *Arabidopsis* (CLV3, CLE8 and CLE13).

Sequence analysis revealed that *FOS1* consists of one exon with a single open reading frame and encodes a putative small protein of 131 amino acids (Figure S1). FOS1 has a signal peptide that is rich in hydrophobic amino acids at its N-terminus and a CLE domain at its C-terminus (Figure 3A). Among the CLE proteins in rice and *Arabidopsis*, the CLE domain of FOS1 is more similar to those of CLE8 and CLE13 than to those of FON2, FCP1 and CLV3 (Figure 3B).

Notably, the nucleotide sequence of *FOS1* in the *fon2-1* mutant (background, Fukei71) was identical to that of standard *japonica* wild-type strains such as Nipponbare and Taichung65 (T65). By contrast, we found an amino acid difference at the putative cleavage site of the signal peptide between *japonica* (all three stains; AB455109) and *indica* (Kasalath; AB455108) (Figure 3A; Figure S1). It is possible that this amino acid substitution in *japonica* FOS1 causes a defect in the processing of FOS1 and a reduction in the amount of active CLE peptide in *japonica*. Except for this mutation, no nucleotide change causing indels of amino acids was detected in the *FOS1* gene between *indica* and *japonica*.

### *FOS1* seems to be involved in meristem maintenance

**Figure 4:**
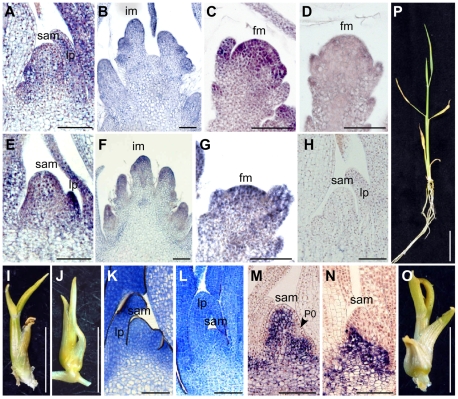
Spatial expression pattern and constitutive expression of *FOS1*. (A–H) In situ localization of *FOS1* transcripts in *indica* (A–D) and *japonica* (E–H): SAM (A,E,H), IM (B,F), and FM (C,D,G). Antisense probe (A–C,E–G), sense probe (D,H). (I, J) Shoots overexpressing *indica FOS1* in wild-type *japonica* (I) and *fon1-5* (J). (K,L) Longitudinal sections of the shoot apex in a shoot expressing control vector (K) and *Act1:indica-FOS1* (L). (M,N) In situ localization of *OSH1* transcripts in a shoot expressing a control vector (M) and *Actin1:indica-FOS1* (N). (O) A shoot overexpressing *japonica FOS1* in wild-type *japonica*. (P) A shoot transformed with a control vector. fm, floral meristem; im, inflorescence meristem; lp, leaf primordia; sam, shoot apical meristem. Scale bars: 100 µm in (A–H,K–N); 5 mm in (I,J,O,P).

We analyzed the spatial and temporal expression patterns of *FOS1* by in situ hybridization. In *indica*, *FOS1* was expressed in all aerial apical meristems, not only in the FM and the IM in the reproductive phase, but also in the SAM in the vegetative phase (Figure 4A–4C). In *japonica*, *FOS1* transcripts were also detected in all apical meristems in a spatial distribution pattern similar to that observed in *indica* (Figure 4E–4G). These spatial expression patterns suggest that FOS1 may be involved in meristem maintenance in rice. In addition to the meristems, *FOS1* transcripts were also detected in the primordia of lateral organs such as the leaf and the floral organs. No significant differences were observed at the transcriptional level in the expression patterns of *FOS1* between *indica* and *japonica*, suggesting that the functional difference between *indica* FOS1 and *japonica* FOS1 is not due to differences at the transcriptional level. No signals were detected in the SAM and FM when sense probes were used (Figure 4D and 4H).

Next, we expressed constitutively *indica FOS1* by using the rice *Actin1* promoter. Unlike shoots transformed with a control vector, *Actin1:indica-FOS1* shoots stopped growing at the seedling stage after a few abnormal and malformed leaves were produced (Figure 4I and 4P). A longitudinal section of the shoot apex revealed that a dome-shaped shoot apical meristem (SAM) was strongly compromised in *Actin1:indica-FOS1* plants, as compared with transgenic seedlings carrying a control vector (Figure 4K and 4L). Next, we examined the expression pattern of rice *OSH1*, an ortholog of *Arabidopsis SHOOT MERISTEMLESS* (*STM*) and maize *knotted1* (*kn1*), which marks undifferentiated cells in the meristem [25]. *OSH1* was expressed uniformly in the meristem except for the site of leaf initiation (P0) (Figure 4M). By contrast, *OSH1* expression was not observed in the meristem of *Actin1:indica-FOS1* shoots (Figure 4N). These results indicate that constitutive expression of *indica-FOS1* terminated meristem function, suggesting that *FOS1* is involved in the maintenance of stem cells in the vegetative SAM as well as in the FM.

### FOS1 is likely to act independently of FON1

To address whether, FON1, a putative receptor of FON2, is required for FOS1 function, we introduced an *indica* genomic fragment containing *FOS1* into the *fon1-5* mutant, which has a severe mutation and is thought to be a null allele of *fon1*. The numbers of floral organs such as stamens and pistils in *fon1-5* transformed with the *indica FOS1* gene were identical to those in wild type, suggesting that *FOS1* functions normally in this *fon1* mutant (Figure 2F–2H). Next, we expressed *indica-FOS1* constitutively in *fon1-5*. The resulting shoot showed a phenotype identical to that of wild type constitutively expressing *indica FOS1* (Figure 4J). These results suggest that the FON1 receptor is not required for the function of FOS1 and that FOS1 is likely to function in an independent signaling pathway.

### Origin of the *japonica FOS1* allele

To elucidate when the mutation observed in *japonica FOS1* occurred during rice evolution, we compared the *FOS1* sequence of a number of varieties/species of domesticated rice and wild rice species such as *O. rufipogon*. To encompass genetic diversity, we examined a core collection of domesticated rice from around the world (WRC, 67 accessions) and from Japan (JRC, 50 accessions) (Table S1; Table S2) [26],[27].

As described above, the one-base change that causes an amino acid substitution at the putative cleavage site of the signal peptide in FOS1 and is associated with its function was found in three *japonica* strains (*fon2-1*, Nipponbare and T65). Hereafter, we call this mutation a functional nucleotide polymorphism (FNP) without reference to the *japonica* or *indica* type. Sequence analysis showed that 66 out of 68 accessions of *japonica* had the FNP (Haplotype B, see below), whereas 59 out of 60 accessions of *indica* did not (Haplotype A) (Table S1; Table S2; Figure S2). Thus, the FNP was closely associated with *japonica* except for three accessions (Calotoc, Pinulupot 1, Padi Perak). Although the accessions in the WRC have been designated *indica* or *japonica* by phenotypic analysis, it seemed likely that the genome of two subspecies might have been introgressed into each other during recent breeding programs. Thus, we examined the type of genome around the *FOS1* locus in the three exceptional accessions by using molecular markers. The results clearly indicated that the FNP is consistent with the *japonica* genome, but not with the *indica* genome (Table 2).

**Table 2:** Genotyping of the chromosomal regions around the *FOS1* locus.

Accession	Classification by phenotype	Genotype							
		Marker	R712	A	B	FNP in FOS1	C	D	R1843
		Location (Mb)	8.9	11.8	12.9	13.0	13.1	14.3	17.8
Calotoc	*indica*		I	J	J	+	J	J	J
Pinulupot 1	*indica*		I	J	J	+	J	J	J
Padi Perek	*japonica*		I	I	I	-	I	I	J

**Figure 5:**
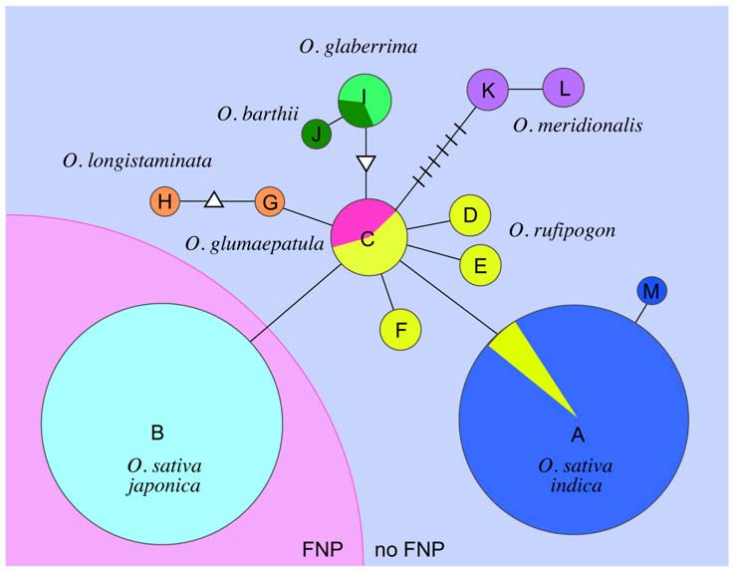
Network of FOS1 haplotypes. The sequences of the haplotypes (A–M) are given in Figure S2. Colors indicate a species or subspecies with the AA genome in the *Oryza* genus. Blue, *O. sativa indica*; light blue, *O. sativa japonica*; yellow, *O. rufipogon*; magenta, *O. glumaepatula*; dark green, *O. barthii*; light green *O. glaberrima*; purple, *O. meridionalis*; orange, *O. longistaminata*. *FOS1* has the FNP in the pink region but does not in the light violet region. The triangle and inverted triangle indicates insertions and deletions, respectively (Figure S2). The hatched line corresponds to a single nucleotide substitution. The number of accessions with each haplotype is roughly indicated by the size of the circle. Accession numbers for the sequences of each haplotype are as follows: FOS1 haplotype A (AB455110), B (AB455111), C (AB455112), D (AB455113), E (AB455114), F (AB455115), G (AB455116), H (AB455117), I (AB455118), J (AB455119), K (AB455120), L (AB455121), and M (AB455122).

Next, we compared the *FOS1* sequence from five wild rice species (22 accessions) and the African domesticated rice *O. glaberrima* (2 accessions), all of which have an AA genome (Table S3). Nucleotide polymorphisms were found in *FOS1* among the wild and domesticated rice accessions (Figure S2). We classified the *FOS1* sequences into 13 haplotypes, and generated a network of these haplotypes (Figure 5). The network indicated that the prototype of *FOS1* is haplotype C, which is shared by two wild rice species, *O. rufipogon* and *O. glumaepatula*. The *FOS1* sequence in wild rice species and domesticated rice may have been derived from this haplotype. None of the accessions of wild rice species showed the FNP at the processing site. Therefore, the FNP in *FOS1* is specific to the genome of *O. sativa japonica.* Because Asian domesticated rice species, namely *japonica* and *indica*, are thought to have derived independently from *O. rufipogon* [28]–[30], this FNP may have occurred during the diversification of *japonica* from *O. rufipogon*.

## Discussion

In this paper, we identified a new *CLE* gene, *FOS1*, in rice by screening for a suppressor of the *fon2* mutation. Like *FON2*, *FOS1* is likely to regulate stem cell maintenance negatively in the FM, but the action of FOS1 is independent of FON1, the putative receptor of FON2. In addition, FOS1 appears to be associated with maintenance of the SAM in the vegetative phase. Genetic analysis suggests that the function of FOS1 in *japonica* appears to be reduced by an FNP occurring at the putative cleavage site of the signal peptide. Distribution of the FNP suggests that this mutation might have occurred during the divergence of *japonica* from its wild ancestor.

### Identification of *FOS1*

The presence of a factor that suppresses *fon* mutations in *indica* was initially assumed from the low expressivity of the *fon* phenotype in F_2_ plants from a cross between *japonica* and *indica*. Although there are two possible explanations for this low expressivity – namely, differences in the genetic background of *japonica* and *indica*, or the presence of a major gene in the genome of *indica* – QTL analysis provided evidence in support of the latter possibility. 

Several lines of evidence suggest that the function of *FOS1* is likely to be compromised in *japonica*. As a result, mutations at the *FON2* locus result in enlargement of the FM and an increase in the floral organ number in *japonica* [8]. In *indica*, by contrast, functional *FOS1* probably masks *fon2* mutations by substituting for *FON2* function in regulating maintenance of the FM (Figure S3). Likewise, in F_2_ plants from a cross between *japonica* and *indica*, *FOS1* derived from *indica* is likely to mask the *fon2* mutation. The frequency (4.5%) of the appearance of the *fon2* phenotype, which is also confirmed by the genotype, in those F_2_ plants is roughly consistent with that expected for the appearance of double mutants. Overexpression of *japonica FOS1* produced an abnormal shoot, as did overexpression of *indica FOS1*, suggesting that *japonica FOS1* is not a complete loss-of-function mutant. In wild-type *japonica*, however, FOS1 CLE peptide, even if produced in part, would be insufficient to restrict stem cells in the FM.

Because our rice research is principally based on *japonica*, *indica FOS1* appears to behave as though it is a suppressor of the *fon2* mutation. A more likely interpretation is, however, that *FOS1* regulates maintenance of the FM redundantly with *FON2* in a wide range of species in the genus *Oryza* (see below) and that *japonica* is a mutant for the *FOS1* locus.

In plant development, it is well known that genes that encode closely related proteins have redundant functions. *APETALA1* (*AP1*) and *CAULIFLOWER* (*CAL*), which that encode MADS-box transcription factors, regulate floral meristem identity together with *LEAFY* [31],[32]. The *ap1 cal* double mutant has a striking phenotype, showing excessive proliferation of the inflorescence meristem, which resembles a cauliflower. This phenotype differs from that of the *ap1* single mutant. Because *CAL* has less effect on floral meristem identity, its single mutation shows no phenotype. *CAL* was identified as an enhancer of the *ap1* phenotype in F_2_ plants from a cross between the *ap1* mutant on a Landsberg *electa* background and wild-type Wassilewskija [31]. Thus, the identification of *FOS1* in this study resembles the discovery of *CAL* in *Arabidopsis*, although *FOS1* has the opposite effect; that is, it appears to be a suppressor of *fon2*. In the case of *AP1* and *CAL*, functional redundancy is due to the factors themselves; by contrast, signaling pathways comprising a different signaling molecule and its receptor might be redundant in the case of meristem maintenance in rice, as discussed below.

### *FOS1* and *FON2* function together in stem cell maintenance in the FM in rice

Our previous study demonstrated that *FON2* is a negative regulator of stem cell maintenance in the FM [8]. In this study, introduction of *indica FOS1* into *fon2-1* by genetic methods using a chromosomal segment substitution line or by *Agrobacterium*-mediated transformation completely suppressed the *fon2* mutation. This finding suggests that *FOS1* can substitute for the function of *FON2*. Thus, *FOS1* is likely to play an important role in maintenance of the FM in *indica* and either one of *FOS1* and *FON2* appears to be sufficient to restrict stem cell proliferation in the FM.

**Figure 6:**
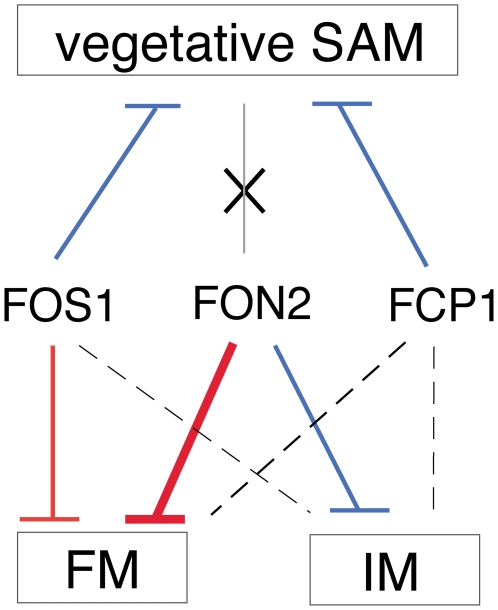
Model of meristem maintenance in rice. Rice is likely to have three negative regulators that restrict stem cell population in the three types of aerial meristem. Each pathway contributes differently depending on the type of meristem. FM, floral meristem; IM inflorescence meristem; Vegetative SAM, shoot apical meristem in the vegetative phase. The pathway indicated by the red thick line has been demonstrated by both loss-of-function and gain-of-function analyses. The pathways indicated by red or blue thin lines have been demonstrated by loss-of-function or gain-of-function analysis, respectively. Dashed lines indicate pathways to be analyzed in future studies. The line marked with X indicates a pathway with no function. See text and our two previous reports for details [8],[24].

There are two possible explanations for the redundancy of FOS1 and FON2. Both CLE peptides may be involved in the same pathway and may share their receptors. Alternatively, there may exist two independent pathways: one involving FOS1 and one involving FON2 as signaling molecules. Two experiments in this study supported the latter possibility. First, a genomic fragment containing *indica FOS1* was able to rescue a severe mutant of *fon1*, in which the putative receptor of FON2 is defective [7]. Second, constitutive expression of *indica FOS1* in *fon1* mutant showed abnormal shoots, a phenotype that is similar to that of wild type overexpressing *indica FOS1*. These results suggest that FON1 is not required for FOS1 function and that the signaling pathways involving FON2 and FOS1 are independent of each other. Because wild species in the *Oryza* genus have no mutation in the functional region of *FOS1*, these two pathways may function in the FM in all *Oryza* species except for *japonica* (Figure 6).

We found that *FOS1* is expressed in the vegetative phase, and constitutive expression of *FOS1* generates abnormal shoots with malformed leaves. It is, therefore, likely that *FOS1* may be involved in maintenance of the vegetative SAM. Constitutive expression of *FON2*, by contrast, does not cause abnormalities in the shoot [24]. In this respect, *FOS1* and *FON2* may have diversified functionally (Figure 6).

In contrast to the FM, the vegetative SAM seems to be unaffected by loss of both *FOS1* and *FON2* because shoot morphology is normal in *fon2* mutants in *japonica* [8]. Therefore, stem cell maintenance in the vegetative SAM may be regulated by an as yet unidentified negative pathway. FCP1 is likely to be involved in this pathway because its constitutive expression consumes stem cells in the vegetative SAM [24]. Thus, stem cell maintenance may be regulated by at least three negative pathways in rice, and each pathway may contribute differently to this regulation depending on the type of the meristem (Figure 6). This situation contrasts with that in *Arabidopsis*, where CLV signaling is the major single pathway in all meristems.

### Distribution of the FNP in FOS1 in the *Oryza* genus

Recent genetic and phylogenetic analyses have revealed that *indica* and *japonica* arose independently from a genetically distinct population in a wild ancestor, *O. rufipogon* [28]–[30],[33]. Our haplotype network of *FOS1* is also consistent with an independent origin of the two subspecies. In our network, haplotype C would have been the prototype of *FOS1* for all domesticated and wild rice species. Haplotype A, associated with *indica*, and haplotype B, associated with *japonica*, would have been produced by the occurrence of a single nucleotide change in haplotype C during rice evolution. *Indica* may have been derived from an *O. rufipogon* species with haplotype A. The FNP at the cleavage site of the signal peptide is responsible for the generation of haplotype B. Although there is no *O. rufipogon* accession with haplotype B, it is possible that *japonica* might have been domesticated from an unidentified ancestor with this FNP.

Many types of mutation are found in *FOS1* of wild rice species, including not only amino acid substitutions but also insertions or deletions (Figure S2). There are, however, no mutations that affect the function of *FOS1* in the coding region, such as an amino acid change in the CLE domain or a frameshift mutation. This observation suggests that defects in *FOS1* may not be neutral and that *FOS1* may be essential for the growth and survival of wild rice species under natural conditions. In line with this hypothesis, it is unlikely that an *O. rufipogon* species that has the FNP in FOS1 will be found in the natural population at present.

## Materials and Methods

### Plant materials

Taichung 65 (T65) and Kasalath were used as representative strains of wild-type *japonica* and *indica*, respectively, in molecular genetic and histochemical analyses. Nipponbare/Kasalath chromosomal segment substitution line #9 (N/K CSSL#9) was obtained from the Rice Genome Resource Center, Japan. Core collections of *O. sativa* (World Rice Collection (WRC) and Japanese Rice Collection (JRC)) were obtained from the Genebank of National Institute of Agrobiological Sciences, Japan (Table S1; Table S2) [26],[27]. Wild rice species were obtained from the National Institute of Genetics, Japan (Table S3).

### QTL analysis and identification of *FOS1*

F_2_ plants from a cross between *fon2-1* and Kasalath were used to search for a gene that suppresses the *fon2* mutation. We obtained 154 F_2_ plants showing a *fon2* phenotype from about 2,000 F_2_ plants and checked their genotypes to confirm that the *fon2* locus has the mutant allele. For QTL analysis, the suppressor activity in each F_2_ plant that had the *fon2* mutation was estimated by calculating the frequency of the twin seeds, an indication of the *fon2* mutation. Next, the genotypes of about 90 loci in the 89 F_2_ plants were determined by using molecular markers [34]. As a result, a major QTL was found at around 40–80 cM on chromosome 2 (LOD score: 6.3). A gene (*FOS1*) encoding a protein with a CLE domain was then identified at around 40 and 80 cM on chromosome 2 by searching the rice genomic sequence database using the amino acid sequence of the FON2 CLE domain as a query.

*FOS1* cDNA was amplified with the appropriate primers (Table S4) from total RNA isolated from young panicles of T65 (*japonica*) and Kasalath (*indica*). After sequencing of the RT-PCR product, the open reading frame was predicted.

### Transformation of rice

To introduce *indica FOS1* into the *fon2* mutant, a 3.3-kb *FOS1* genomic fragment, including 2.6 kb of sequence directly upstream of the initiation codon of *FOS1*, from the Kasalath genome was used. For constitutive expression of *FOS1*, a *FOS1* cDNA derived from T65 or Kasalath was placed under the rice *Actin1* promoter [35]. The resulting plasmids, designated *Actin1:japonica-FOS1* (T65) and *Actin1:indica-FOS1* (Kasalath), were introduced into *Agrobacterium tumefaciens* strain EHA101 and transformed into rice as described previously [36].

### In situ hybridization

For the in situ hybridization probe for *FOS1*, a 646-bp fragment consisting of the entire coding region, the 5′ UTR (137 bp) and the 3′ UTR (113 bp) was amplified with the appropriate primers (Table S4). The fragment was cloned into a T-vector by TA-cloning (Novagen, Madison). The *OSH1* probe was prepared as described in the original paper [25]. Probe synthesis, preparation of sections, in situ hybridization, and microscopic observation were performed as described previously [7],[24].

### Analysis of FOS1 haplotype

The genomic region of *FOS1* was amplified with the appropriate primers (Table S4). The amplified fragments were purified with Montage PCR Filter Units (Millipore, Billerica) and sequenced with the same primers used for amplification. The haplotype network was constructed by using the program TCS1 [37].

## Supporting Information

Figure S1 Sequences of the *FOS1* gene and FOS1 protein. (A) Nucleotides sequence corresponding to the coding region of the *indica FOS1* gene. The nucleotide substituted in *japonica* is indicated in red. (B) Amino acid sequence of the *indica* FOS1 protein. The amino acid substituted in *japonica* is indicated in red. Amino acids in the putative active CLE peptide are indicated in blue. Underline indicates the putative signal sequence predicted by signalP (http://www.cbs.dtu.dk/services/SignalP) [1].
(0.02 MB PDF)

Figure S2 Nucleotide changes or indels in *FOS1* haplotypes. The FNP is located at the 69th position. Haplotype C is used as a reference. Synonymous substitution is indicated with light blue and nonsynonymous substitution with pink. Twelve nucleotides are deleted at positions 85–96 without a frameshift in haplotype H. Positions 358–393 correspond to the CLE domain (12 aa).
(0.05 MB PDF)

Figure S3 FON2 and FOS1 redundantly restrict stem cell proliferation in the FM in *indica* but not in *japonica*. See text for details.
(0.00 MB PDF)

Table S1 Accessions in WRC and their *FOS1* haplotypes.
(0.02 MB PDF)

Table S2 Accessions in JRC and their *FOSI* haplotypes.
(0.01 MB PDF)

Table S3 Accessions of wild rice species and African domesticated rice and their *FOS1* haplotypes.
(0.01 MB PDF)

Table S4 Primers use in this study.
(0.03 MB PDF)
