# Supplementary material for: FON2 SPARE1 Redundantly Regulates Floral Meristem Maintenance with FLORAL ORGAN NUMBER2 in Rice
Source: PLoS Genet. 2009 Oct 16;5(10):e1000693. doi: 10.1371/journal.pgen.1000693 (PMC2752996; doi:10.1371/journal.pgen.1000693)
Supplement: Figure S1 — Sequences of the FOS1 gene and FOS1 protein. (A) Nucleotides sequence corresponding to the coding region of the indica FOS1 gene. The nucleotide substituted in japonica is indicated in red. (B) Amino acid sequence of the indica FOS1 protein. The amino acid substituted in japonica is indicated in red. Amino acids in the putative active CLE peptide are indicated in blue. Underline indicates the putative signal sequence predicted by signalP (http://www.cbs.dtu.dk/services/SignalP) [1]. (0.02 MB PDF) [file pgen.1000693.s001.pdf]

A

ATGAGCCGGCGACTCGGCGCCGCGGCCGCCGTCCTCCTCCTGTGGCTCGCCGTGCTCACC

TTCGCCTTCCACGGCTACTACGGCGGCCGCCTCGGCTCCGCGAGGCGGCGGAATATCCTC

└→ G

CTCCAACACCCGGCGCTAGCGCTCCATCTCCCCACCAGGAAGATGCTCCTCGCCGTGGCG

AGCTTCGACGACGCCTCCTCGCCGTCGTCGTTGACGACCACCGATCGTCATCATCACCAT

CACAGGCATCATGGCCACCACCATCATCGCGGCCATGATCGGTGGAACAGGAAGGGTGTC

CCGCCGACGGCAGCTGGGCCGGGCGAGGAGGTCGACCCGCGGTTCGGCGTGCAGAAGAGG

CTGGTCCCGACGGGCCCCAACCCCCTGCACCATTGA

B

MSRRLGAAAAVLLLWLAVLTFAFHGYYGGRLGSARRRNILLQHPALALHLPTRKMLLAVA

└→ L

SFDDASSPSSLTTTDRHHHHHRHHGHHHHRGHDRWNRKGVPPTAAGPGEEVDPRFGVQKR

LVPTGPNPLHH
