# Supplementary material for: FON2 SPARE1 Redundantly Regulates Floral Meristem Maintenance with FLORAL ORGAN NUMBER2 in Rice
Source: PLoS Genet. 2009 Oct 16;5(10):e1000693. doi: 10.1371/journal.pgen.1000693 (PMC2752996; doi:10.1371/journal.pgen.1000693)
Supplement: Figure S2 — Nucleotide changes or indels in FOS1 haplotypes. The FNP is located at the 69th position. Haplotype C is used as a reference. Synonymous substitution is indicated with light blue and nonsynonymous substitution with pink. Twelve nucleotides are deleted at positions 85–96 without a frameshift in haplotype H. Positions 358–393 correspond to the CLE domain (12 aa). (0.05 MB PDF) [file pgen.1000693.s002.pdf]

	Haplotype	Number of accessions	8	27	**69**	100	125	150	179	204	216	230	252	262	273	279	314	320	deletion
O.sativa indica	**A**	57	G	C	**C**	G	/	C	C	A	G	A	T	C	C	T	C	C	
O. rufipogon	**A**	3	G	C	**C**	G	/	C	C	A	G	A	T	C	C	T	C	C	
O. sativa japonica	**B**	67	G	C	**G**	G	/	C	C	G	G	A	T	C	C	T	C	C	
O. rufipogon	**C**	4	G	C	**C**	G	/	C	C	G	G	A	T	C	C	T	C	C	
O. glumaepatula	**C**	3	G	C	**C**	G	/	C	C	G	G	A	T	C	C	T	C	C	
O. rufipogon	**D**	2	G	C	**C**	A	/	C	C	G	G	A	T	C	C	T	C	C	
O. rufipogon	**E**	2	G	C	**C**	G	/	C	T/C	G	G	A	T	C	C	T	C	C	
O. rufipogon	**F**	2	G	C	**C**	G	/	C	C	G	G	A	T	T	C	T	C	C	
O. longistaminata	**G**	1	G	C	**C**	G	/	C	C	G	G	G	T	C	C	T	C	C	
O. longistaminata	**H**	1	G	C	**C**	G	/	C	C	G	G	G	T	C	C	T	C	C	12bp (85-96)
O. glaberrima	**I**	2	G	C	**C**	G	TCC	C	C	G	G	A	T	C	C	T	C	C	
O. barthii	**I**	1	G	C	**C**	G	TCC	C	C	G	G	A	T	C	C	T	C	C	
O. barthii	**J**	1	C	C	**C**	G	TCC	C	C	G	G	A	T	C	C	T	C	C	
O. meridionalis	**K**	1	G	C	**C**	G	/	C	C	G	A	A	C	C	G	C	T	A	
O. meridionalis	**L**	1	G	C	**C**	G	/	T	C	G	A	A	C	C	G	C	T	A	
O. sativa indica	**M**	1	G	T	**C**	G	/	C	C	A	G	A	T	C	C	T	C	C	
