# Supplementary figures and images for: FON2 SPARE1 Redundantly Regulates Floral Meristem Maintenance with FLORAL ORGAN NUMBER2 in Rice
Source: PLoS Genet. 2009 Oct 16;5(10):e1000693. doi: 10.1371/journal.pgen.1000693 (PMC2752996; doi:10.1371/journal.pgen.1000693)

indica

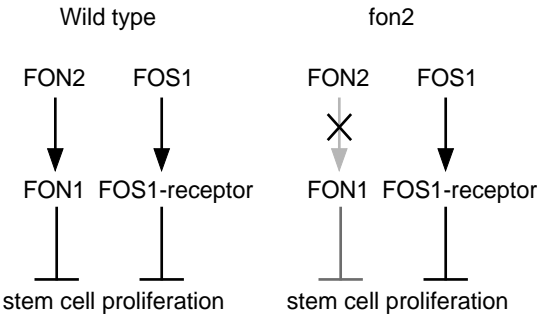

japonica

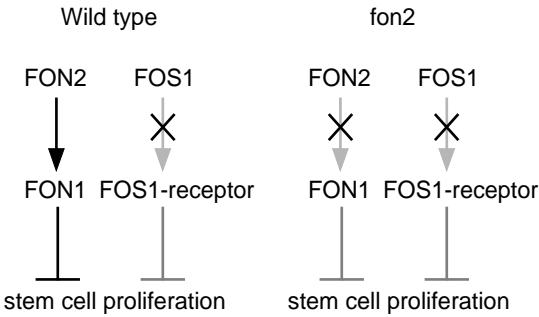

FM size

normal

normal

normal

increased

Supplement: Figure S3 — FON2 and FOS1 redundantly restrict stem cell proliferation in the FM in indica but not in japonica. See text for details. (0.00 MB PDF) [file pgen.1000693.s003.pdf]
