# Supplementary material for: FON2 SPARE1 Redundantly Regulates Floral Meristem Maintenance with FLORAL ORGAN NUMBER2 in Rice
Source: PLoS Genet. 2009 Oct 16;5(10):e1000693. doi: 10.1371/journal.pgen.1000693 (PMC2752996; doi:10.1371/journal.pgen.1000693)
Supplement: Table S1 — Accessions in WRC and their FOS1 haplotypes. (0.02 MB PDF) [file pgen.1000693.s004.pdf]

Table S1. Accessions in WRC and their *FOS1* haplotypes

WRC No.	Accessions	Origin	*FOS1* haplotype	Classification by phenotype	Genotype around the *FOS1* locus*
1	NIPPONBARE	Japan	B	japonica	B
2	KASALATH	India	A	indica	A
3	BEI KHE	Cambodia	A	indica	A
4	JENA 035	Nepal	A	indica	A
5	NABA	India	A	indica	A
6	PULUIK ARANG	Indonesia	A	indica	A
7	DAVAO 1	Philippines	A	indica	A
9	RYO SUISAN KOUMAI	China	A	indica	A
10	SHUUSOUSHU	China	A	indica	A
11	JINGUOYIN	China	A	indica	A
13	ASU	Bhutan	A	indica	A
14	IR 58	Philippines	A	indica	A
15	CO 13	India	A	indica	A
16	VARY FUTSI	Madagascar	A	indica	A
17	KEIBOBA	China	A	indica	A
18	QINGYU	China	A	indica	A
19	DENG PAO ZHAI	China	A	indica	A
20	TADUKAN	Philippines	A	indica	A
21	SHWE NANG GYI	Myanmar	A	indica	A
22	CALOTOC	Philippines	B	indica	A
23	LEBED	Philippines	M	indica	M
24	PINULUPOT 1	Philippines	B	indica	A
25	MUHA	Indonesia	A	indica	A
26	JHONA 2	India	A	indica	A
27	NEPAL 8	Nepal	A	indica	A
28	JARJAN	Bhutan	A	indica	A
29	KALO DHAN	Nepal	A	indica	A
30	ANJANA DHAN	Nepal	A	indica	A
31	SHONI	Bangladesh	A	indica	A
32	TUPA 121-3	Bangladesh	A	indica	A
33	SURJAMUKHI	India	A	indica	A
34	ARC 7291	India	A	indica	A
35	ARC 5955	India	A	indica	A
36	RATUL	India	A	indica	A
37	ARC 7047	India	A	indica	A
38	ARC 11094	India	A	indica	A
39	BADARI DHAN	Nepal	A	indica	A
40	NEPAL 555	India	A	indica	A
41	KALUHEENATI	Sri Lanka	A	indica	A
42	LOCAL BASMATI	India	A	indica	A
43	DIANYU 1	China	B	japonica	B
44	BASILANON	Philippines	A	indica	A
45	MA SHO	Myanmar	B	japonica	B
46	KHAO NOK	Laos	B	japonica	B
47	JAGUARY	Brazil	B	japonica	B
48	KHAU MAC KHO	Vietnam	B	japonica	B
49	PADI PERAK	Indonesia	A	japonica	B
50	REXMONT	USA	B	japonica	B
51	URASAN 1	Japan	B	japonica	B
52	KHAU TAN CHIEM	Vietnam	B	japonica	B
53	TIMA	Bhutan	B	japonica	B
55	TUPA 729	Bangladesh	B	japonica	B
57	MILYANG 23	Korea	A	indica	A
58	NEANG MENH	Cambodia	A	indica	A
59	NEANG PHTONG	Cambodia	A	indica	A
60	HAKPHAYNHAY	Laos	A	indica	A

61	RADIN GOI SESAT	Malaysia	A	indica	A
62	KEMASIN	Malaysia	A	indica	A
63	BLEIYO	Thiland	A	indica	A
64	PADI KUNING	Indonesia	A	indica	A
65	RAMBHOG	India	A	indica	A
66	BINGALA	Myanmar	A	indica	A
67	PHULBA	India	B	japonica	B
68	KHAO NAM JEN	Laos	B	japonica	B
97	CHIN GALAY	Myanmar	A	indica	A
98	DEEJIAOHUALUO	China	A	indica	A
99	HONG CHEUH ZAI	China	A	indica	A
100	VANDARAN	Sri Lanka	A	indica	A

* See Table 2 and the text.
