# Supplementary material for: FON2 SPARE1 Redundantly Regulates Floral Meristem Maintenance with FLORAL ORGAN NUMBER2 in Rice
Source: PLoS Genet. 2009 Oct 16;5(10):e1000693. doi: 10.1371/journal.pgen.1000693 (PMC2752996; doi:10.1371/journal.pgen.1000693)
Supplement: Table S2 — Accessions in JRC and their FOSI haplotypes. (0.01 MB PDF) [file pgen.1000693.s005.pdf]

Table S2. Accessions in JRC and their *FOS1* haplotypes

JRC No.	Accessions	Classification by phenotype	Haplotype
1	GAISEN MOCHI	*japonica*	B
3	HINODE	*japonica*	B
4	SENSHOU	*japonica*	B
5	YAMADA BAKE	*japonica*	B
6	KANEKO	*japonica*	B
7	IRIMA NISHIKI	*japonica*	B
8	OKKA MODOSHI	*japonica*	B
10	HIRAYAMA	*japonica*	B
11	KAHEI	*japonica*	B
12	OIRAN	*japonica*	B
13	BOUZU MOCHI	*japonica*	B
14	MEGURO MOCHI	*japonica*	B
17	AKAGE	*japonica*	B
18	MASOKUHO	*japonica*	B
19	WATARIBUNE	*japonica*	B
20	HOSOGARA	*japonica*	B
21	AKAMAI	*japonica*	B
22	MANSAKU	*japonica*	B
23	ISHIJIRO	*japonica*	B
24	JOUSHUU	*japonica*	B
25	DANGO	*japonica*	B
26	AIKOKU	*japonica*	B
27	GINBOUZU	*japonica*	B
28	SHINRIKI MOCHI	*japonica*	B
29	SHICHIENCHOU MOCHI	*japonica*	B
30	MORITA WASE	*japonica*	B
31	KAMEJI	*japonica*	B
32	OMACHI	*japonica*	B
33	SHINRIKI	*japonica*	B
34	KYOUTOASAHI	*japonica*	B
35	KABASHIKO	*japonica*	B
36	SEKIYAMA	*japonica*	B
37	SHINYANADAHO 2	*japonica*	B
38	NAGOYA SHIRO	*japonica*	B
39	SHIROINE (KEMOMI)	*japonica*	B
40	AKAMAI	*japonica*	B
41	AKAMAI	*indica*	A
42	TOUBOSHI	*indica*	A
43	AKAMAI	*indica*	A
44	KARAHOUSHI	*indica*	A
45	HIYADACHITOU	*japonica*	B
46	FUKOKU	*japonica*	B
47	OKABO	*japonica*	B
48	HAKAMURI (YOKOHAMA)	*japonica*	B
49	RIKUTOU RIKUU 2	*japonica*	B
50	HIMENOMOCHI	*japonica*	B
51	SHINSHUU	*japonica*	B
52	AICHIASAHI	*japonica*	B
53	RANDEN	*japonica*	B
54	HOUMANSHINDEN INE	*japonica*	B
