# Supplementary material for: FON2 SPARE1 Redundantly Regulates Floral Meristem Maintenance with FLORAL ORGAN NUMBER2 in Rice
Source: PLoS Genet. 2009 Oct 16;5(10):e1000693. doi: 10.1371/journal.pgen.1000693 (PMC2752996; doi:10.1371/journal.pgen.1000693)
Supplement: Table S3 — Accessions of wild rice species and African domesticated rice and their FOS1 haplotypes. (0.01 MB PDF) [file pgen.1000693.s006.pdf]

Table S3. Accessions of wild rice species and african domesticated rice and their *FOS1* haplotypes.

Species	Accessions	Origin		Haplotype
O. rufipogon	W0106	India	1958	A
O. rufipogon	W1866	Thailand	1984	A
O. rufipogon	W2263	Cambodia	2001	A
O. rufipogon	W1236	Australian New Guinea	1961	C
O. rufipogon	W1807	Sri Lanka	1981	C
O. rufipogon	W1945	China	1984	C
O. rufipogon	W2078	Australia	1993	C
O. rufipogon	W1294	Philippines	1963	D
O. rufipogon	W2003	India	1985	D
O. rufipogon	W0120	India	1958	E
O. rufipogon	W2051	Bangladesh	1990	E
O. rufipogon	W0630	Burma	1960	F
O. rufipogon	W1921	Thailand	1984	F
O. glumaepatula	W1169	Cuba	1960	C
O. glumaepatula	W2145	Brazil	1994	C
O. glumaepatula	W2199	Brazil	1996	C
O. longistaminata	W1413	Sierra Leone	1964	G
O. longistaminata	W1508	Madagascar	1964	H
O. glaberrima	C7599	Nigeria	1959	I
O. glaberrima	C8538(B)	Guinea	1963	I
O. barthii	W1588	Cameroun	1978	I
O. barthii	W0652	Sierra Leone	1960	J
O.meridionalis	W1625	Australia	1978	K
O.meridionalis	W1635	Australia	1978	L
