# Supplementary material for: FON2 SPARE1 Redundantly Regulates Floral Meristem Maintenance with FLORAL ORGAN NUMBER2 in Rice
Source: PLoS Genet. 2009 Oct 16;5(10):e1000693. doi: 10.1371/journal.pgen.1000693 (PMC2752996; doi:10.1371/journal.pgen.1000693)
Supplement: Table S4 — Primers use in this study. (0.03 MB PDF) [file pgen.1000693.s007.pdf]

Table S4. Primers used in this study

Screenig of the plants homozygous for the *fon2* mutation			Restriction Enzyme
	F2 d1	5'-TGTGTTTGCTTGACATGGGC-3'	
	F2 u2	5'-GCCGCCTCATCCAGAGCA-3'	
Screenig of the plants homozygous for the *FOS1* gene of *indica*			
	FOSK d7	5'-GCTGCTGATGGAGACGAATT-3'	NdeI
	F2S u2	5'-CTAGCTAGCCTTTGGTCGAT-3'	
FOS1 RT-PCR			
	F2S d1	5'-CTTGCCGCCGTGCTTAGCTT-3'	
	F2S u2	5'-CTAGCTAGCCTTTGGTCGAT-3'	
Construct for *fon1* complementation			
	FOSK d1	5'-TCATTGTTGTTGTCTCACCC-3'	
	FOSK u2	5'-ATAGTGATCAGTTCGGCTTA-3'	
FOS1 in situ hybridization			
	F2S d1	5'-CTTGCCGCCGTGCTTAGCTT-3'	
	F2S u2	5'-CTAGCTAGCCTTTGGTCGAT-3'	
Construct for *Actin:FOS1*			
	ActFOS2 d1-Xb	5'-GATCTAGAAGCCTGCAGCGCGGTGGA-3'	
	ActFOS2 u2-Xb	5'-GATCTAGAACTCGAGCACCCATCAAT-3'	
FOS1 sequence			
	F2S d1	5'-CTTGCCGCCGTGCTTAGCTT-3'	
	F2S u2	5'-CTAGCTAGCCTTTGGTCGAT-3'	
Genotyping around *FOS1* locus			
R712	R712 d1	5' -ATGGCATGAGTTTGGGGAAG- 3'	
	R712 u2	5' -AGGCACTTGTCAGATGGTTC- 3'	
A	1185 d1	5'-AATAGGATCAAAGAGGCAAA-3'	XspI
	1185 u2	5'-GGCAAGAAGAGAAGCAATAG-3'	
B	1296 d1	5'-AACGCGGTCAGATCACTAGA-3'	AluI
	1296 u2	5'-GTTTCGGCACCTATTTTTGT-3'	
C	1316 d1	5'-TACATTCAGCACAGGACCTC-3'	MboI
	1316 u2	5'-AGTTCCTTATGGTATTTCCG-3'	
D	1446 d1	5'-GTTGTAATCATGCTTGTCGA-3'	SphI
	1446 u2	5'-ATTTGATCTACAGGAGGCTT-3'	
R1843	R1843 d1	5' -TCCAGGCGCTTCTTTGATGC- 3'	
	R1843 u2	5' -ATCGCGGCAACACCTCAAAG- 3'	
